# Factors influencing implementation dose and fidelity thereof and related student outcomes of an evidence-based national HIV prevention program

**DOI:** 10.1186/s13012-015-0236-y

**Published:** 2015-04-07

**Authors:** Bo Wang, Bonita Stanton, Lynette Deveaux, Maxwell Poitier, Sonja Lunn, Veronica Koci, Richard Adderley, Linda Kaljee, Sharon Marshall, Xiaoming Li, Glenda Rolle

**Affiliations:** 1 Department of Pediatrics, Wayne State University School of Medicine, 4707 Saint Antoine Street, Detroit, MI 48201 USA; 2 Office of HIV/AIDS, Ministry of Health, Shirley Street, Nassau, Bahamas; 3 Department of Education, Ministry of Education, Thompson Boulevard, PO Box N-3913, Nassau, Bahamas

**Keywords:** Implementation cluster, HIV prevention, Implementation dose, Fidelity of implementation, Adolescents, The Bahamas

## Abstract

**Background:**

Teachers’ implementation of evidence-based prevention programs in schools is inconsistent. Using data gathered from the national implementation among grade six students in The Bahamas of an evidence-based HIV intervention [Focus on Youth in the Caribbean (FOYC)], this study examines differences in the degree of implementation (“dose”) and adherence to the core activities (“fidelity of implementation”) by teachers according to theoretically and historically relevant teachers’ characteristics, attitudes, and experiences pre-intervention and post-intervention. The relationship of implementation dose and implementation fidelity is assessed according to student outcomes.

**Methods:**

Beginning in 2008, the Bahamian Ministry of Education (MOE) included FOYC in the grade six curriculum nationwide. Consistent with standard practice, teachers were offered MOE training workshops in FOYC prior to delivery. The MOE conducted an anonymous curricular assessment among the grade six students at the beginning and end of the school year. Teachers agreeing to participate in the research component were asked to complete a pre-implementation and post-implementation assessment of attitudes and prior experiences.

**Results:**

Teachers taught 15.6 out of 30 core activities, 24 out of the 46 total activities, and 4.6 out of 8 sessions on average. Three teachers’ implementation groups were identified: 1) High Implementation Group (31.7% of the teachers), characterized by high levels of implementation dose and fidelity of implementation; 2) Moderate Implementation Group (52.8%), showing moderate levels of implementation dose but high levels of fidelity of implementation; and 3) Low Implementation Group (15.6%), with low levels of implementation dose and fidelity of implementation. Low Implementation Group teachers compared to teachers in the two higher performing groups had less training in interactive teaching, limited prior exposure to the FOYC curriculum, incomplete attendance at FOYC training workshops, and low levels of comfort in teaching FOYC lessons. Students taught by teachers in the Low Implementation Group demonstrated poorer outcomes relevant to the four student outcomes (HIV/AIDS knowledge, preventive reproductive health skills, self-efficacy, and intention to use protection if they were to have sex).

**Conclusions:**

Both implementation dose and implementation fidelity are related to student outcomes. Teachers at risk for limited implementation can be identified pre-intervention, thus opening the possibility for focused pre-intervention training.

## Introduction

Evidence-based prevention and health promotion interventions offer great promise for improving the well-being of a population. Accordingly, much progress has been made in developing and evaluating health-promoting interventions addressing a range of disorders yielding a substantial portfolio of effective public-health programs. By contrast, less progress has been made in assuring their introduction and sustained use in an effective manner in the community [1]. The past decades have witnessed the development of robust HIV prevention intervention portfolios as illustrated by the Center for Disease Control’s identification of 44 “best evidence” HIV prevention programs through its “Prevention Synthesis Project” [2]. Despite significant declines in AIDS-related deaths and lower rates of new HIV infection, the potential impact of evidence-based behavioral prevention programs has been muted because of the difficulties in sustaining effective delivery of these interventions in real-life settings as opposed to effectiveness trials. For interventions addressing children and adolescents, school-based programs are especially appealing given their wide reach to a “captive” audience. Initiating implementation of effective programs within a school system is a critically important and complex first step and sustaining these efforts even more so, and sustaining them with fidelity has proven to be a daunting task [3-6].

Sustainability of evidence-based interventions has been identified as a major research priority area for many years, producing some instructive findings [7,8]. The extant literature indicates that sustained implementation of evidence-based behavioral interventions is low [6]. Factors reported to influence sustainability of implementation within a school system include the following: program’s acceptability and perceived relative importance to school administrators and teachers, perceived effectiveness of the intervention, feasibility to implement on an ongoing basis, and flexibility and adaptability [9-13].

Studies assessing fidelity of implementation of effective programs (using a range of definitions, criteria, and cutoffs) likewise find “low” levels of fidelity [14]. Factors associated with *fidelity* of implementation include ease of integration of the program into their existing curricular approach/routine [8,15] and teacher training in the curriculum [16-18]. Teacher characteristics found to be related to fidelity include the following: teacher’s positive attitude towards prevention programs [19,20], shorter duration of time as a teacher [6], and confidence in one’s ability to teach interactive methods [6]. Teacher perceptions that are important include the following: the intervention reflects the values of and is “from” their community [21], students’ are engaged in the intervention curriculum, and students are positively impacted by the intervention [15,22].

There is evidence that the more similar the intervention delivery is to that which was demonstrated to be effective, the more likely it is that positive outcomes will be realized. A recent systematic review reveals that a majority of studies have found that higher implementation dose and/or implementation fidelity are associated with better program outcomes [23]. Blakely and colleagues conducted a comprehensive examination of fidelity and effectiveness assessing seven nationally disseminated education and criminal justice projects and found that implementations conducted with high-fidelity were more effective than low-fidelity implementations [24]. Other studies have found that suboptimal quality delivery of evidence-based programs can result in minimal or null effects [14]. An evaluation of 14 school-based anti-bullying programs found that the majority of programs yielded non-significant outcomes on measures of self-reported victimization and bullying [25]. Derzon et al. assessed intervention effectiveness of drug prevention programs and found no significant effect on drug use among program participants [26]. These studies suggest that inadequate program delivery may overshadow the potential impact of prevention programs [25,26]. However, Blakely’s research noted above [24] did suggest that if adaptation of the curriculum occurs in the form of local *additions* to the program, effectiveness appeared to be increased or at least not decreased, supporting the view that *some* adaptation may be important in enabling appropriate fit of the intervention in a new setting.

The literature systematically identifying implementation patterns by teachers of effective HIV prevention programs in school settings is sparse, but some relevant studies do exist. Klingner et al. categorized teachers who participated in the implementation of four research-based programs into three implementation groups [27], and Shin et al. identified five teachers’ delivery profiles based on teacher engagement and delivery techniques [28]. While informative, these studies are limited by the qualitative nature of data, the modest sample sizes, and the non-systematic methods for identification of the implementation clusters. The current study seeks to fill this research gap by identifying teachers’ implementation patterns using cluster analysis and relating implementation patterns to student outcomes.

Understanding whether and why teachers did and did not implement an intervention as it was designed is important. Critical to improving outcomes is the ability to identify teachers who are at increased risk of not implementing an intervention in a manner likely to bring greatest benefit before teaching the curriculum. Therefore, in the present analyses, we sought to address four questions relevant to implementation of an effective HIV risk prevention program. First, do teachers vary in their degree of implementation of an HIV risk reduction intervention in terms of implementation dose (e.g., how much of the intervention do they deliver) and integrity of implementation (e.g., do they adhere to the activities and presentations as described in the intervention manual)? Second, are groups of teachers who vary in their degree of implementation identifiable prior to intervention delivery? If so, what are the characteristics of these groups? Third, do these characteristics change in response to intervention delivery? Fourth, do student outcomes vary according to either or both implementation dose and fidelity of implementation?

## Methods

### Study site

From 2011 through 2013, all 80 government elementary schools in the Commonwealth of The Bahamas participated in national implementation of Focus on Youth in the Caribbean (FOYC), including the research component described herein. The 80 schools are located on 14 of the major islands constituting The Bahamas, where more than 98% of the population resides. Forty-six schools were located on the three most populated islands: 25 schools on Island #1 (I-1), 11 schools on Island #2 (I-2), and 10 schools on Island #3 (I-3). The remaining 34 schools were located on eleven small islands. The 80 participating schools housed 208 grade six classes and teachers: I-1 housed 122 (58.7% of the total) teachers; I-2, 27 (13%) teachers; I-3, 16 (7.7%) teachers; and the six small islands housed 43 (20.7%) teachers. The research protocol was approved by the Wayne State University Human Investigation Committee and the Institutional Review Board of the Bahamian Princess Margaret Hospital, Public Hospitals Authority.

### HIV prevention program

FOYC was adapted from Focus on Youth (FOY), a ten-session sexual risk reduction program targeting mid-adolescents. FOY and Informed Parents and Children Together (CImPACT) are currently part of the Centers for Disease Control and Prevention’s “Diffusion of Effective Behavioral Interventions (DEBI)” Portfolio. FOYC and FOY are based on a social cognitive model, the Protection Motivation Theory [29]. The intervention includes games, interactive discussions, stories, and exercises to reinforce main messages and increase knowledge and skills regarding sexual risk avoidance, effective communication, negotiation, and condom use [30]. Multiple exercises in communication and a decision-making model is applied in most of the sessions illustrating the steps that should guide a child in selecting a course of action. Intervention evaluations showed that the intervention significantly increased Bahamian youth’s HIV/AIDS knowledge, perceptions of their ability to use condoms, and condom use intention [31,32] with evidence of increased condom use [33].

### Teacher training

The Bahamian Ministry of Education (MOE) launched 11 teacher training workshops following the protocol used by the DEBI program to train future interventionists in the delivery of FOY including two 4-day-, eight 2-day-, and one 1-day-long workshops. Duration was based on available time in the MOE workshop training schedules. Regardless of the length of the workshop, the teacher training covered the following: 1) review of the need for HIV prevention in The Bahamas, 2) overview of FOYC including the research showing its effectiveness, 3) a walk-through of each of the sessions of FOYC with participation and “role-play” of the core activities considered to be critical to the success of FOYC, and 4) a didactic question-and-answer period regarding contraception and condom use. The longer workshops (4 days compared to 2 days compared to 1 day) provided a more detailed curriculum experience, longer didactic session, and more teacher involvement in role-plays and discussions. All 208 teachers (regardless of attendance at a workshop) were given a copy of the FOYC teacher training manual.

Nearly half of the teachers (49.2%) attended some or all of a FOYC training workshop in 2011/2012, 30.6% completed the workshop, and 23 (11%) teachers reported that they had attended a FOYC workshop before 2010 when they participated in the original FOYC intervention effectiveness trial. Overall, 60.2% of the teachers received training supporting their delivery of the FOYC curriculum, with the remainder receiving no training.

### Measures

#### Implementation dose and fidelity of implementation

To assess implementation, all teachers were asked to complete a Teacher Implementation Checklist specific for each of the eight sessions of FOYC after they had taught the session. The checklist includes all 46 activities in the FOYC curriculum, 30 of which were identified by the developers as “core elements”. The teachers indicated which activities they had and had not taught in each session; *implementation dose* was defined as the number of the 30 core activities that were taught. For those core activities that they taught, the teachers recorded whether they had modified the format of the activity as outlined in the manual to determine *fidelity of implementation*. Teachers also recorded the total number of *all activities* (from among 46) that they taught. The number of sessions taught was calculated by summing the sessions in which a teacher taught one or more core activities; if teachers taught any core activity within a session, it counted as “teaching that session”.

#### Factors associated with implementation: teachers’ perceptions of the FOYC curriculum content, their prior training experience, and other factors shown to impact intervention implementation and fidelity

At pre- and post-intervention delivery, all participating teachers were asked to complete questionnaires assessing factors described in the prior research summarized in the “Introduction” section as influencing fidelity of intervention implementation. Both pre-implementation and post-implementation questionnaires assessed the following: teachers’ perceptions of the importance of prevention programs, HIV prevention, and FOYC intervention (very important, somewhat important, or not important) for grade six students in their community or schools; teacher’s confidence in teaching the FOYC intervention; teacher’s sense of “ownership” of the curriculum (i.e., perceiving it as a “Bahamian intervention”); and the relative importance of their time spent in teaching FOYC compared to the time spent teaching reading skills in grade six (less important, about the same, more important) [6,14,19,21].

The *pre-implementation* questionnaire also collected information on the teacher’s level of formal education, years as a teacher/guidance counselor, teacher’s attendance at FOYC training workshop, training in interactive teaching, and prior experience of teaching FOYC or other HIV prevention programs. The *post-implementation* questionnaire further assessed the role of competing priorities as a reason for not being able to complete delivering the FOYC curriculum and perceived student benefits from FOYC curriculum (very much, somewhat/not at all). Hypothetical relationships among these variables are discussed in a prior publication [34].

In bivariate analyses, responses were grouped into two or three collapsed categories due to low frequencies in some categories: years as a teacher/guidance counselor (1–5 years, 6–10 years, >10 years), training in interactive teaching (a lot/some, a little/none), perceptions of the importance of HIV prevention (very important and somewhat/not important), and confidence in teaching FOYC (very comfortable and somewhat/not comfortable). The Teacher Implementation Checklist described above asked teachers to record their degree of comfort in teaching the lesson (very comfortable, rather comfortable, or not comfortable) and how many students (most, some, few) appeared to be engaged in the lesson.

#### Student outcomes

An anonymous curricular assessment instrument (with identifying information only at the level of the school and teacher), adapted by the MOE from a version of the Bahamian Youth Health Risk Behavioral Inventory (BYHRBI) [30], was administered by the MOE to grade six students at the beginning of the grade six before receipt of FOYC and at the end of grade six. The instrument assessed HIV/AIDS knowledge and preventive reproductive health skills, as well as some perceptions, intentions, and self-reported behaviors. A 15-item scale including true and false statements was used to assess the level of *HIV/AIDS knowledge*. The internal consistency of the scale was high (Cronbach’s *α* = 0.85). Correct responses were scored 1 and incorrect 0, resulting in a summary score of 0 to 15 for each participant. *Preventive reproductive health skills* were assessed through an adaptation of the Condom-use Skills Checklist [35]. The validated scale includes true and false statements describing the steps of correct condom use from opening a condom pack for use to disposal after use. This six-item scale demonstrated adequate internal consistency (Cronbach’s *α* = 0.83). Correct responses were scored 1 and incorrect 0, resulting in a summary score of 0 to 6 for each participant. A three-item scale was used to assess *self-efficacy* for using pregnancy/STI prevention methods. All three items employed a yes/no response scale, with one point assigned for each “yes” response. Individual item scores were added to yield a summary score (range 0 to 3). The internal consistency of the scale was 0.81. *Intention to use protection* was measured using the question, “if you were to have sex in the next six months, how likely is it that you would use a condom (to prevent yourself from getting HIV)?” Youth rated the likelihood on a five-point Likert scale ranging from 1 (very unlikely) through 5 (very likely).

### Analysis

Cluster analysis was used to identify teachers who were at high risk for inadequate implementation FOYC *before* they taught the course. Cluster analysis is a person-oriented approach which identifies homogenous groups within a sample of diverse individuals based on similarities across a set of attributes [36]. A two-step cluster analysis in SPSS 22 based on the two variables was used to define implementation dose (i.e., number of core activities completed) and fidelity of implementation (i.e., percentage of core activities being changed during the implementation). The optimal number of clusters was determined automatically by the statistical software based on Schwarz’s Bayesian information criterion, and the overall quality of the cluster solution was investigated based on the Silhouette measure of cohesion and separation and each variable’s level of importance to the cluster solution [37].

The differences in teachers’ education, total years as a teacher, attendance of training workshop, training in interactive training, prior experience of teaching FOYC or other HIV prevention programs, and perceptions of importance of HIV prevention or FOYC to grade six youth between the clusters were examined using chi-square test. The number of all activities completed and number of sessions taught in the classroom were compared across the clusters using ANOVA test.

Teachers’ post-implementation perceptions of the importance of HIV prevention, program ownership, student benefits from FOYC intervention, comfort level in teaching intervention curriculum, relative importance of the time spent in teaching FOYC and teaching reading skills, competing priorities, and student engagement were compared across the clusters, using chi-square test (for categorical variables) and ANOVA test (for continuous variables). Differences in teachers’ perceptions of the importance of HIV prevention, program ownership, and comfort level in teaching FOYC at pre-intervention and post-implementation were assessed to determine if these factors changed during the course of teaching the curriculum using McNemar’s test.

To assess differential intervention effects on student outcomes (HIV/AIDS knowledge, preventive reproductive health skills, self-efficacy, intention to use protection) by teachers’ implementation cluster membership, the change over time within each group of students, as well as the differences in change scores between the clusters, were compared using the Student *t* test and ANOVA with the Tukey honestly significant difference *post hoc* tests. The association of teachers’ patterns of implementation (High, Moderate, and Low Implementation Groups, with the Low Implementation Group serving as the reference group) with student outcomes was examined using mixed-effects modeling, adjusting for clustering effects of classroom and/or school. Independent variables included teachers’ implementation clusters (high/moderate/low implementers), student’s age, sex, and baseline student outcomes. School and class were included as random effect variables in the model. Regression coefficients were calculated for all variables. All the analyses except the two-step cluster analysis were performed using SAS 9.3 statistical software package (SAS Institute Inc., Cary, NC, USA).

## Results

### Implementation dose and fidelity of implementation of FOYC intervention

**Figure 1:**
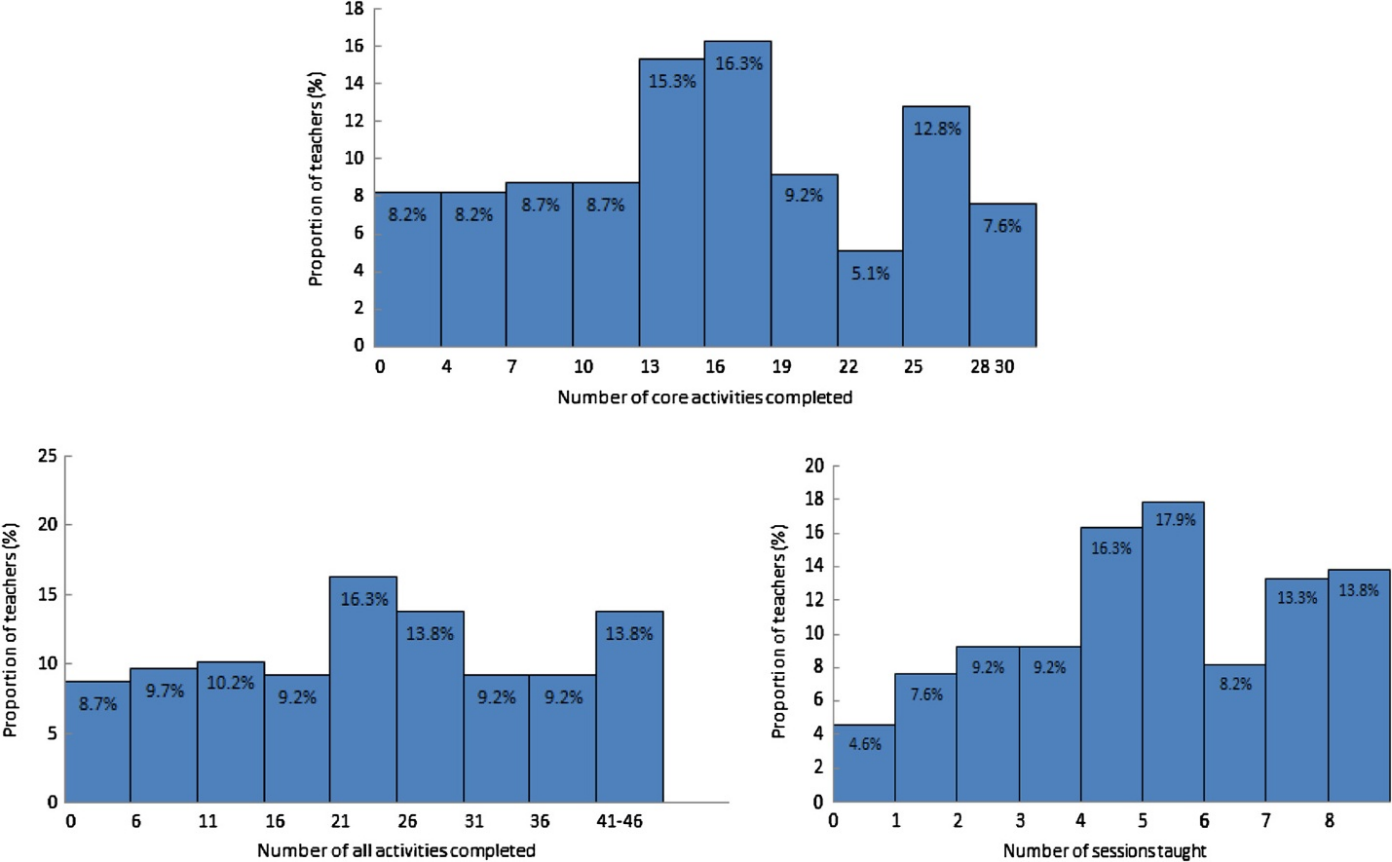
**Number of core activities, all activities, and sessions in the FOYC curriculum taught by 208 teachers.**

The number of core activities, “all activities” (including core and non-core activities), and sessions taught by 208 teachers are displayed in Figure 1. On average, teachers taught 15.6 core activities (SD = 8.2) from among the 30 core activities, 24 of the 46 total activities (“all activities”) (SD = 12.8), and 4.6 sessions (SD = 2.3) from among the total eight sessions. Only two (1%) teachers completed all core activities and covered all eight sessions while six (2.9%) teachers did not teach any activities in their classes. Sixteen (7.7%) teachers taught ≥28 core activities; 28 (13.5%) taught ≥40 core and non-core activities; 56 (27%) taught seven or eight sessions of FOYC curriculum. Overall, 38 (18.3%) teachers taught less than eight core activities (and less than two sessions). Teachers changed 3.1 core activities (SD = 3.7) on average. Nearly one third of teachers did not change any core activities while one third changed one to three core activities. Approximately 10% of teachers changed ≥10 core activities.

### Teachers’ implementation groups

Results of the two-step cluster analysis indicated three distinct clusters or groups of teachers. The Silhouette measure of cohesion and separation was above 0.5, indicating good cluster quality [38]. The predictor importance indicated that both the number of core activities completed (i.e., implementation dose) and percentage of core activities being changed (i.e., fidelity of implementation) are very important for the clustering solution (score range: 0.9–1.0). The first cluster of teachers, which includes 31.7% of the teachers, is characterized by the highest probabilities of teaching core activities in the classroom and thus is labelled the “High Implementation Group”. Teachers in this cluster taught over 80% of core activities (25 out of 30 core activities) on average and changed only 14% of the core activities. The second cluster, the “Moderate implementation Group”, is the largest cluster (52.8% of the teachers) and is distinguished by moderate probabilities of teaching core activities when compared with the High implementation Group. Teachers in the Moderate Implementation Group taught less than half of core activities (12.3 out of 30 core activities) on average and changed 13% of core activities that they taught in the classroom, similar to the rate of change of activities in the High Implementation Group. The third cluster, the “Low Implementation Group”, is the smallest cluster (15.6%) and is characterized by a combination of the lowest probabilities of teaching core activities and higher probabilities of modifying core activities compared to the other two groups; these taught less than one third of core activities (8.6 out of 30 core activities) on average and changed three quarters of core activities that they taught in the classroom.

Teachers’ implementation group membership was also related to numbers of *all activities* completed in the classroom. Teachers in the High Implementation Group taught the greatest numbers of all activities, followed by teachers in the Moderate Implementation Group, with teachers in the Low Implementation Group having taught the lowest numbers of all activities (38.9 vs. 18.9 vs. 12.6, *p* < 0.001). Teachers in the High Implementation Group also taught more sessions than teachers in the Moderate and Low Implementation Groups (7.2 vs. 3.6 vs. 3.0, *p* < 0.001) (Table 1).

**Table 1:** **Teacher patterns of implementation and pre-implementation characteristics, teaching and training experiences and attitudes towards HIV prevention**

Variables	Overall	Clusters				
		High Implementation Group	Moderate Implementation Group	Low Implementation Group	*χ* ^2^	*p*
Cluster sizes		31.7%	52.8%	15.6%		
Number of core activities taught		25.14	12.33	8.65		
Proportion of core activities changed		14%	13%	76%		
Education level						
Associate degree/teaching certificate	13.0%	12.1%	15.1%	5.6%	5.99	0.2000
Bachelor degree	73.4%	81.0%	66.3%	83.3%		
Master degree	13.6%	6.9%	18.6%	11.1%		
Total years as a teacher or guidance counselor						
1–5 years	16.9%	13.6%	20.5%	10.5%	15.98	0.0030
6–10 years	25.9%	42.4%	13.6%	31.6%		
>10 years	57.2%	44.1%	65.9%	57.9%		
Attendance of training workshop						
Did not attend training workshop	50.8%	46.0%	52.4%	54.8%	4.92	0.0226
Attended part of a workshop	18.6%	14.3%	14.3%	41.9%		
Fully attended a training workshop	30.6%	39.7%	33.3%	3.2%		
Training in interactive teaching						
A little/none	41.6%	27.1%	43.2%	79.0%	16.10	0.0003
A lot/some	58.4%	72.9%	56.8%	21.0%		
Prior experience of teaching FOYC						
No	86.1%	81.4%	88.6%	89.5%	1.77	0.4132
Yes	13.9%	18.6%	11.4%	10.5%		
Prior experience of teaching other HIV prevention programs						
No	77.7%	83.1%	78.4%	57.9%	5.30	0.0705
Yes	22.3%	16.9%	21.6%	42.1%		
Importance of HIV prevention for grade 6 youth in general						
Somewhat important/not at all	13.4%	15.2%	12.6%	11.1%	0.30	0.8613
Very important	86.6%	84.8%	87.4%	88.9%		
Importance of Focus on Youth for grade 6 youth in your school						
Somewhat important/not at all	12.1%	6.8%	16.1%	10.5%	2.91	0.2330
Very important	87.9%	93.2%	83.9%	89.5%		
Comfort level in teaching the FOYC lessons						
Somewhat/not at all	43.7%	34.5%	42.4%	77.8%	10.43	0.0054
Very comfortable	56.3%	65.5%	57.6%	22.2%		
Islands						
Capital island	60.8%	73.0%	58.1%	45.2%	7.45	0.0241
Other family islands	39.2%	27.0%	41.9%	54.8%		
FOY curriculum is a Bahamian curriculum						
Somewhat/not at all	43.4%	45.4%	42.5%	41.2%	0.16	0.9254
Very much	56.6%	54.6%	57.5%	58.8%		
Compared to the time spent teaching reading skills in grade six, the time spent teaching FOYC was						
Less important	16.2%	20.4%	11.3%	28.6%	3.71	0.1564
About the same/more important	83.8%	79.6%	88.7%	71.4%		
Number of sessions taught (1–8 sessions)^a^	4.57 (2.33)	7.22 (0.79)	3.63 (1.48)	3.03 (2.20)	144.35	<0.0001
Number of all activities completed (0–46 activities)^a^	23.75 (12.73)	38.94 (4.38)	18.92 (7.58)	12.58 (9.41)	207.41	<0.0001

Note: ^a^*F* test was used.

### Association of teachers’ implementation group membership with teachers’ characteristics and pre- and post-implementation perceptions

Table 1 shows the differences between the three groups of teachers in terms of pre-implementation characteristics, teaching and training experiences, and attitudes towards HIV prevention. Duration of experience as a teacher, training in interactive teaching, attendance of the FOYC training workshop, comfort level in teaching the FOYC lessons, and islands where the teachers worked significantly differed among the High, Moderate, and Low Implementation Groups. More teachers in the Moderate or Low Implementation Groups than teachers in the High Implementation Group had worked as a teacher or guidance counselor for over 10 years (66% vs. 58% vs. 44%, *χ*^2^ = 15.98, *p* < 0.01). In contrast, more teachers in the High Implementation Group had served as a teacher for 6 to 10 years. Higher proportions of teachers in the High and Moderate Implementation Groups compared to teachers in the Low Implementation Group received extensive training in interactive teaching (73% vs. 57% vs. 21%, *χ*^2^ = 16.10, *p* < 0.001), indicated high levels of comfort in teaching FOYC lessons (66% vs. 58% vs. 22%, *χ*^2^ = 10.43, *p* < 0.01), and attended all aspects of their training workshops (40% vs. 33% vs. 3%, *χ*^2^ = 4.92, *p* < 0.05). Although the workshops differed by length, teachers completing their assigned training did not differ significantly in terms of implementation dose or fidelity of implementation. More teachers in the High Implementation Group were teaching in Island #1 (the most populous Bahamian island and the island in which FOYC was originally adapted for The Bahamas and tested for efficacy) (73% vs. 58% vs. 45%, *χ*^2^ = 7.45, *p* < 0.05) while more teachers in the Low Implementation Group worked in the other islands constituting The Bahamas (the “Family Islands”). Teachers’ level of education, prior experiences of teaching FOYC or other HIV prevention programs, program ownership, and perceptions of importance of HIV prevention for grade six youth were not significantly different among the three implementation groups.

Of the eight perceptions assessed post-intervention, five differed significantly between the three implementation groups: perceived importance of FOYC, program priority, perceived student benefits, ownership of the FOYC curriculum, and comfort level in teaching core activities. More teachers in the High Implementation Group perceived that the FOYC intervention was very important for grade six students *in their schools*, their students benefited very much from the intervention, and the FOYC curriculum was a Bahamian intervention. More teachers in the Moderate and Low Implementation Groups than teachers in the High Implementation Group reported that they had other priorities than teaching FOYC in their daily work. Teachers in the High and Moderate Implementation Groups reported higher levels of comfort in teaching core activities in the FOYC curriculum than teachers in the Low Implementation Group. Teachers’ perceptions regarding the importance of HIV prevention for grade six youth in general and student engagement in FOYC lessons were not associated with teachers’ implementation group membership (Table 2).

**Table 2:** **Teacher patterns of implementation and post-implementation perceptions, program ownership, perceived student benefit, and comfort level in teaching intervention curriculum**

		Clusters				
Variables	Overall	High Implementation Group	Moderate Implementation Group	Low Implementation Group	*χ* ^2^	*p*
Cluster sizes		31.7%	52.8%	15.6%		
Importance of HIV prevention for grade 6 youth in general						
Somewhat important/not at all	10.3%	5.1%	13.6%	11.1%	2.81	0.2460
Very important	89.7%	94.9%	86.4%	88.9%		
Importance of Focus on Youth for grade 6 youth in your school						
Somewhat important/not at all	17.8%	3.5%	20.9%	40.0%	17.20	0.0002
Very important	82.2%	96.5%	79.1%	60.0%		
Having other priorities (than teaching FOYC)						
No	64.3%	84.1%	57.1%	48.4%	16.56	0.0003
Yes	35.7%	15.9%	42.9%	51.6%		
Perceived benefits of FOY curriculum for grade 6 students						
Somewhat/not at all	23.7%	6.8%	25.0%	57.7%	26.04	<0.0001
Very much	76.3%	93.2%	75.0%	42.3%		
FOY curriculum is a Bahamian curriculum						
Somewhat/not at all	41.0%	27.6%	41.0%	72.0%	14.25	0.0008
Very much	59.0%	72.4%	59.0%	28.0%		
Compared to the time spent teaching reading skills in grade six, the time spent teaching FOYC was:						
Less important	17.7%	10.2%	22.7%	18.2%	3.82	0.1482
About the same/more important	82.3%	89.8%	77.3%	81.8%		
Student engagement in core activities (range 1–3 points)^a^	2.85 (0.23)	2.87 (0.21)	2.87 (0.19)	2.79 (0.29)	1.82	0.1648
Comfort level in teaching core activities (range 1–3 points)^a^	2.82 (0.24)	2.87 (0.16)	2.83 (0.24)	2.74 (0.30)	3.13	0.0459

Note: ^a^*F* test was used.

### Changes in teachers’ perceptions between pre-intervention and post-intervention

Almost all teachers (97%–98.8%) perceived that prevention programs or HIV prevention programs were very important for youth pre-intervention. Higher proportions of teachers in the High and Moderate Implementation Groups than teachers in the Low Implementation Group perceived that prevention programs were very important for youth post-intervention (100% vs. 98% vs 89%, *χ*^2^ = 8.54, *p* < 0.05) while the rates were comparable pre-intervention. The majority of teachers indicated that HIV prevention was very important for grade six youth in general or FOYC was very important for grade six youth in their school. Comparable at baseline, the proportion of teachers who post-intervention perceived the FOYC to be of great importance for grade six youth in their school was highest in the High Implementation Group, followed in order by the Moderate Implementation Group and the Low Implementation Group (97% vs. 79% vs 60%, *χ*^2^ = 17.20, *p* < 0.001). Within-group comparison indicated that the proportion of teachers who perceived the importance of FOYC in the Low Implementation Group was reduced from 90% pre-intervention to 60% post-intervention (*χ*^2^ = 4.73, *p* < 0.05). Over 80% of the teachers perceived that their time spent teaching FOYC is about the same or more important than time spent teaching reading skills in grade six; this perception did not differ significantly pre-intervention and post-intervention or across the three groups. Nearly 60% of the teachers perceived the FOYC curriculum as a Bahamian intervention (“ownership”). Higher proportions of teachers in the High and Moderate Implementation Groups than teachers in the Low Implementation Group perceived program ownership post-intervention (72% vs. 59% vs. 28%, *χ*^2^ = 14.25, *p* < 0.01) while the rates had been comparable pre-intervention. The proportion of teachers who perceived program ownership in the Low Implementation Group reduced from 59% pre-intervention to 28% post-intervention (*χ*^2^ = 3.99, *p* < 0.05) while it increased from 54% pre-intervention to 72% post-intervention for teachers in the High Implementation Group (*χ*^2^ = 3.90, *p* < 0.05). Comfort level in teaching FOYC increased from pre-intervention to post-intervention for teachers in all three groups. Comfort level in teaching FOYC for teachers in the Low Implementation Group was lower than that of teachers in the High Implementation Group post-intervention (2.9 vs. 2.8 vs. 2.7, *F* = 3.13, *p* < 0.05) while the rates were comparable pre-intervention.

### Teachers’ implementation group membership and student outcomes

Table 3 presents the change in HIV/AIDS knowledge, preventive reproductive health skills, self-efficacy, and intention to use protection from baseline to follow-up among students according to the implementation group membership of their teacher. All student outcomes increased significantly among all three teacher implementation groups over the 12 months. At baseline, knowledge and reproductive health skills were higher among students whose teachers belonged to the High or Moderate Implementation Groups. At follow-up, knowledge and reproductive health skills were highest among students whose teachers belonged to the High Implementation Group, followed in order by students whose teachers were in the Moderate Implementation Group and students whose teachers were in the Low Implementation Group (knowledge: 10.5 vs. 10.0 vs. 9.2, *F* = 58.21, *p* < 0.001; skills: 4.0 vs. 3.7 vs. 3.6, *F* = 27.13, *p* < 0.001). At baseline, self-efficacy and intention to use protection were comparable across the three implementation groups. At follow-up, self-efficacy and intention were significantly higher among students whose teachers were in the High or Moderate Implementation Groups compared to students whose teachers were in the Low Implementation Group (self-efficacy: 1.3 vs. 1.3 vs. 1.1, *F* = 9.17, *p* < 0.001; intention: 3.3 vs. 3.2 vs. 2.9, *F* = 10.43, *p* < 0.001); students whose teachers were in the High or Moderate Implementation Groups demonstrated greater increases in self-efficacy and intention than students whose teachers were in the Low Implementation Group.

**Table 3:** **Teacher patterns of implementation and student outcomes (*n*** **= 4,411)**

		Clusters				
Variables	Overall	High Implementation Group	Moderate Implementation Group	Low Implementation Group	*F*	*p*
HIV/AIDS knowledge (range 0–15 points)						
Baseline	8.22 (2.62)	8.59 (2.50)	8.18 (2.58)	7.83 (2.59)	21.75	<0.0001
Follow-up	9.95 (2.55)	10.45 (2.24)	9.98 (2.52)	9.17 (2.66)	58.21	<0.0001
Increase (follow-up-baseline)	1.73 (2.58)	1.86 (2.38)	1.79 (2.56)	1.33 (2.62)	9.95	<0.0001
Preventive reproductive health skills (range 0–6 points)						
Baseline	3.37 (1.31)	3.54 (1.28)	3.38 (1.30)	3.07 (1.33)	27.09	<0.0001
Follow-up	3.77 (1.27)	3.98 (1.17)	3.73 (1.29)	3.56 (1.36)	27.13	<0.0001
Increase (follow-up-baseline)	0.40 (1.29)	0.44 (1.22)	0.35 (1.29)	0.48 (1.34)	3.27	0.038
Self-efficacy (range 0–3 points)						
Baseline	0.78 (1.05)	0.79 (1.04)	0.80 (1.05)	0.77 (1.09)	0.22	0.8064
Follow-up	1.23 (1.17)	1.32 (1.16)	1.26 (1.19)	1.08 (1.11)	9.17	0.0001
Increase (follow-up-baseline)	0.45 (1.11)	0.53 (1.10)	0.46 (1.12)	0.31 (1.10)	8.10	0.0002
Intention to use protection (range 1–5 points)						
Baseline	2.38 (1.73)	2.47 (1.77)	2.35 (1.72)	2.39 (1.73)	1.96	0.1411
Follow-up	3.17 (1.79)	3.27 (1.77)	3.22 (1.79)	2.87 (1.82)	10.43	<0.0001
Increase (follow-up-baseline)	0.79 (1.76)	0.79 (1.77)	0.86 (1.75)	0.48 (1.77)	9.95	<0.0001

The results of the mixed-effects models indicate that teachers’ patterns of implementation were significantly related to improvement in all four student outcome measures. At follow-up, compared to students whose teachers belonged to the Low Implementation Group, students whose teachers were in the High and/or Moderate Implementation Groups demonstrated higher levels of HIV/AIDS knowledge, reproductive health skills, self-efficacy, and intention to use protection if they were to engage in sex after controlling for age, gender, baseline difference, and clustering effects of school and classroom. Older age was associated with improvement in condom use self-efficacy. Male gender was associated with increased reproductive health skills, self-efficacy, and intention to use protection. Classroom random effects were significant in all four models, indicating significant variation among classrooms with regard to students’ knowledge of HIV/AIDS, reproductive health skills, self-efficacy, and intention to use protection. School random effects were significant for self-efficacy only (Table 4). The mixed-effects models were rerun using the Moderate Implementation Group as the reference group. Compared to students whose teachers belonged to the Moderate Implementation Group, students whose teachers were in the High Implementation Group demonstrated higher levels of HIV/AIDS knowledge (*β* = 0.48, SE = 0.18, *t* = 2.64, *p* = 0.008) and reproductive health skills (*β* = 0.24, SE = 0.07, *t* = 3.46, *p* = 0.0005).

**Table 4:** **Mixed-effects models assessing the association between teacher’s pattern of implementation and students’ outcomes**

Variables	Estimated models											
	HIV/AIDS knowledge			Preventive reproductive health skills			Self-efficacy			Intention to use protection		
	*β*	SE	*t*	*β*	SE	*t*	*β*	SE	*t*	*β*	SE	*t*
*Fixed effect*												
Intercept	8.557	0.617	13.87^***^	3.046	0.334	9.11^***^	1.013	0.282	3.59^***^	2.794	0.484	5.77^***^
Age	0.041	0.051	0.79	0.029	0.029	0.99	0.164	0.025	6.68^***^	−0.021	0.042	−0.49
Gender												
Male	−0.051	0.078	−0.66	0.147	0.043	3.41^***^	0.553	0.037	14.87^***^	0.529	0.062	8.60^***^
Female (ref)												
Baseline student outcome	0.019	0.016	1.21	0.017	0.017	1.03	−0.036	0.018	−2.02^*^	0.016	0.018	0.90
Teacher’s clusters												
High Implementation Group	1.236	0.264	4.68^***^	0.489	0.103	4.76^***^	0.198	0.099	2.00^*^	0.400	0.169	2.37^*^
Moderate Implementation Group	0.752	0.252	2.98^**^	0.245	0.098	2.50^*^	0.157	0.094	1.67	0.335	0.161	2.08^*^
Low Implementation Group												
*Random effect*												
School^a^	-	-		0.007	0.011	0.65	0.049	0.016	3.07^**^	0.048	0.032	1.48
Class (nested within school)^a^	0.936	0.130	7.18^***^	0.082	0.020	4.14^***^	0.037	0.012	3.10^***^	0.219	0.047	4.69^***^

*p < 0.05; **p < 0.01; ***p < 0.001. ^a^*z* test.

## Discussion

That teachers’ cluster into three identifiable groups according to their levels of implementation dose and implementation fidelity and that these groups are significantly associated with student performance outcomes are important findings. Nearly one third of the teachers were identified as high implementers. These teachers taught over 80% of core activities in their classroom and adhered to the format for 86% of the activities outlined in the manual. Low implementers, representing approximately one sixth of the teachers, taught less than one third of core activities and modified most of these taught. About half of the teachers were identified as moderate implementers; these teachers taught less than half of all core activities but they adhered to the format of most of the activities that they taught.

Teachers’ implementation group membership is associated with both prior teaching experience and intervention training experience. Teachers in the High Implementation Group were less likely to have been teaching for more than 10 years than teachers in the other two groups and were more likely to have received training in interactive teaching. These findings are consistent with previous research suggesting that fewer years of teaching experience and confidence in using interactive methods in intervention delivery are positively associated with fidelity of implementation [6,39].

Teachers’ full attendance in the FOYC training workshop is associated with High (and Moderate) Implementation Group membership. By contrast, no training or attending only part of the assigned workshop is associated with Low Implementation Group membership. Duration of training was not in and of itself the important factor. This observation is not consistent with previous research suggesting that longer training is associated with higher quality implementation [18]. Rather, *full* attendance was the critical component, suggesting that a workshop can be streamlined provided that the critical components of the intervention are retained in the training. This is important in long-term implementation, as multi-day training programs are expensive for government-supported educational systems and difficult for teachers to accommodate in their schedules.

Teachers’ High Implementation Group membership is associated with teachers’ perceptions of the importance of the FOYC intervention for grade six students, student benefits from the intervention, and program ownership at post-intervention, which are consistent with prior studies [5,14,19,21]. Pre-intervention perceptions regarding the importance of FOYC and program ownership did not differ by implementation group membership; pre-intervention, all three groups expressed endorsement of the importance of such educational material for students. Teachers in the High Implementation Group retained this perspective, while those in the Moderate Implementation Group experienced some erosion and those in the Low Implementation Group experienced a marked decrease of this endorsement. This finding suggests that something occurred in the latter two groups to undermine their confidence in the curriculum. Whether this decrease resulted in the sub-optimal implementation or their sub-optimal implementation resulted in their changed perception is not known but requires further exploration as potential remedies will vary. It is noteworthy that teachers in the High Implementation Group did not perceive a conflict in terms of other priorities competing with teaching FOYC. Whether this reflects actual differences in the scheduled courses or other obligations of teachers in the High and Low Implementation Groups or it reflects differing perspectives requires further evaluation as implications for intervening will differ.

Teachers in the Low Implementation Group exhibited several distinguishing characteristics pre-intervention compared to teachers in the two higher performing clusters: less training in interactive teaching, limited prior exposure to the FOYC curriculum, failure to attend the full training FOYC workshop, and low levels of comfort in teaching FOYC lessons. Lack of prior training experience and low levels of comfort in teaching intervention activities may increase teachers’ anxiety which in turn has an impact on implementation quality, particularly when prevention programs are perceived as competing with other priorities [40]. Missing part of a training workshop may contribute to low-quality implementation and/or may reflect disinterest or discomfort (or both) with the curriculum. These warning signs are easily identifiable pre-intervention or during early implementation and should alert the need for additional training or support. More investigation is needed to better understand the reasons for and resolutions to inadequacies in implementation of FOYC. In the interim, strategies could include increasing teachers’ awareness of adolescent risk behaviors and health consequences, engaging in discussions with teachers about the importance of HIV prevention programs in their schools/communities, reviewing the data with teachers regarding curriculum impact on student outcomes, and enhancing teachers’ competency in teaching the intervention curriculum.

Regardless of teachers’ implementation clusters, students’ HIV/AIDS knowledge, reproductive health skills, self-efficacy, and intention to use protection significantly increased from baseline to follow-up. These changes are similar to those found in previous randomized controlled trials [31,32]. Our study reveals that quality of implementation was significantly related to student outcomes (better implementation leads to better outcomes). Students whose teachers were in the High and/or Moderate Implementation Groups demonstrated greater improvements in three student outcomes (knowledge, skills, self-efficacy, and intention) compared to students whose teachers belonged to the Low Implementation Group. Students whose teachers were in the High Implementation Group demonstrated greater improvements in HIV/AIDS knowledge and reproductive health skills compared to students whose teachers belonged to the Moderate Implementation Group. These findings are consistent with previous research suggesting that implementation dose and implementation fidelity influences program outcomes [23].

There are several potential limitations in this study. First, our findings are based on teachers’ self-reports of their extent of implementation of the FOYC intervention. It is possible that teachers over-reported their level of implementation and provided responses that they thought would be more appropriate. In the current study, trained observers independently observed and assessed approximately 20% of each teacher’s classes. The teacher and observer reports on activities covered in these sessions were compared to determine the level of agreement; in general, we found that the observer-teacher agreement was high (over 80%), indicating that teachers’ self-reports of their implementation of the intervention curriculum in their classrooms are reliable. Second, school-level factors such as support by the principal and school administrator’s perception of importance of HIV intervention were only collected from about half of the participating schools. Thus, these data were not included in the present analysis.

Our study adds to the sparse but emerging literature on the implementation of evidence-based interventions in school settings. Findings regarding inconsistent implementation, three teachers’ implementation groups, and differences in teacher characteristics, teaching, and training experiences and pre- and post-implementation perceptions by implementation groups provide a greater understanding of the barriers and facilitators impacting large-scale implementation of effective intervention programs in schools. The results suggest that additional training or assistance should be provided to those teachers who received minimum training in interactive teaching and in teaching the intervention curriculum, who were not confident in teaching intervention curriculum or held less favorable attitudes towards intervention programs at pre-intervention. Finally, while the questions asked pre-intervention clearly identify at-risk teachers, additional information is needed to identify remedies to the sub-optimal teaching performance. Findings from this study can help interventionists or health practitioners develop better approaches to promote the implementation of effective HIV prevention interventions in schools.
